# 

**Published:** 1886-10

**Authors:** 


					﻿Iiole Numbar, 321.
-OCTOBER, I886.
Vol. LI 11. Number 4.
THE
HICAGO MEDICAL JOURNAL AND EXAMINER
EDITORS:
S. J. JONES, M.D., LL.D.
W. JAGGARD, A.M., M.D.
HAROLD N. MOYER, M.D.
PUBLISHED MONTHLY BY
THE CLABK & LOKGLEY CO.,
Under direction of
THE CHICAGO MEDICAL PRESS ASSOCIATION,
308-316 Deareorn Street.
				

